# Amyloid Disassembly: What Can We Learn from Chaperones?

**DOI:** 10.3390/biomedicines10123276

**Published:** 2022-12-17

**Authors:** Zaida L. Almeida, Rui M. M. Brito

**Affiliations:** Chemistry Department and Coimbra Chemistry Centre—Institute of Molecular Sciences (CQC-IMS), University of Coimbra, 3004-535 Coimbra, Portugal

**Keywords:** protein misfolding, protein aggregation, aberrant aggregates, amyloid fibrils, amyloidosis, amyloid disassembly, disaggregases, molecular chaperones, chemical chaperones, pharmacological chaperones

## Abstract

Protein aggregation and subsequent accumulation of insoluble amyloid fibrils with cross-β structure is an intrinsic characteristic of amyloid diseases, i.e., amyloidoses. Amyloid formation involves a series of on-pathway and off-pathway protein aggregation events, leading to mature insoluble fibrils that eventually accumulate in multiple tissues. In this cascade of events, soluble oligomeric species are formed, which are among the most cytotoxic molecular entities along the amyloid cascade. The direct or indirect action of these amyloid soluble oligomers and amyloid protofibrils and fibrils in several tissues and organs lead to cell death in some cases and organ disfunction in general. There are dozens of different proteins and peptides causing multiple amyloid pathologies, chief among them Alzheimer’s, Parkinson’s, Huntington’s, and several other neurodegenerative diseases. Amyloid fibril disassembly is among the disease-modifying therapeutic strategies being pursued to overcome amyloid pathologies. The clearance of preformed amyloids and consequently the arresting of the progression of organ deterioration may increase patient survival and quality of life. In this review, we compiled from the literature many examples of chemical and biochemical agents able to disaggregate preformed amyloids, which have been classified as molecular chaperones, chemical chaperones, and pharmacological chaperones. We focused on their mode of action, chemical structure, interactions with the fibrillar structures, morphology and toxicity of the disaggregation products, and the potential use of disaggregation agents as a treatment option in amyloidosis.

## 1. Introduction

**Figure 1:**
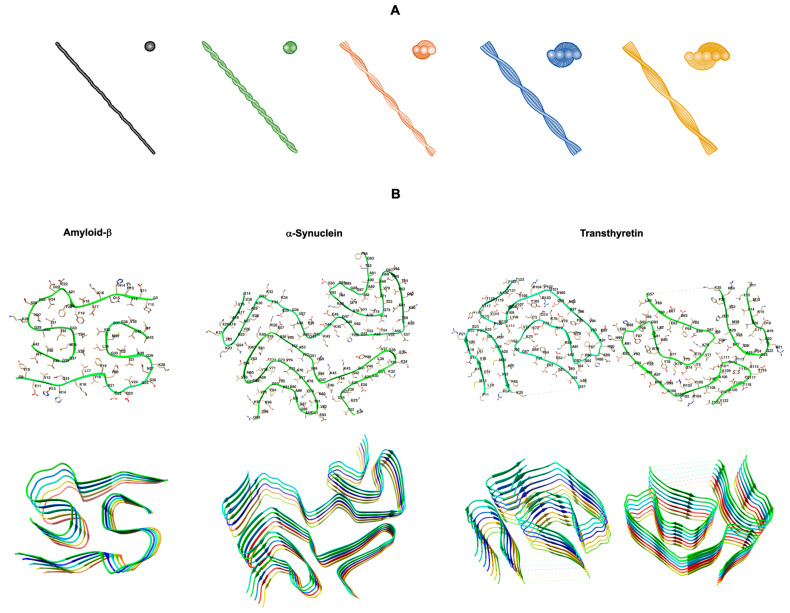
Structural polymorphism of amyloid fibrils. (**A**) 3D morphology models of reconstructed amyloid fibrils with different number of protofilaments: one (black), two (green), three (orange), four (blue), and five (yellow) protofilaments. Adapted from [2]. (**B**) Cryo-electron microscopy structures of the core regions of amyloid protofilaments of amyloid-β 1-42 (Aβ42) (PDB code: 7Q4B) [3], α-synuclein (PDB code: 6XYO) [4], and transthyretin (TTR) (PDB code: 6SDZ) [5] from patients with sporadic Alzheimer’s disease, sporadic synucleinopathy, and hereditary Val30Met TTR amyloidosis, respectively. The (**B**) upper panel displays the backbone and side chains of one dimer pair, while the (**B**) lower panel illustrates the cross-β structure of stacked dimers along the fibril axis. Structures produced with UCSF Chimera [6] using the respective PDB codes.

Protein misfolding and aggregation are key events in a large group of progressive, degenerative, and highly debilitating diseases [1]. The ability of a linear polypeptide chain to fold correctly into its native tridimensional conformation and perform its physiological function is the basic dogma in structural biology and it is essential for the survival of living organisms. However, the free energy difference between unfolded and folded states of proteins is fairly small, and thus misfolded states are readily accessible to polypeptide chains in the presence of the right environmental conditions or covalent structure modifications. These may include gene mutations, chemical modifications of the native protein, partial hydrolysis of the polypeptide chain, and changes in pH, physiological conditions or extracellular, biomembrane, or intracellular molecular partners. These misfolded and partially unfolded states in general expose larger hydrophobic surfaces, which lead to protein aggregation into amorphous aggregates or highly ordered β-sheet rich amyloid oligomers and fibrils. The accumulation of amyloid fibrils, insoluble supramolecular assemblies with fibrillar morphology and cross-β structure (Figure 1), constitutes the hallmark of well-known amyloid pathologies, including the neurodegenerative Alzheimer’s, Parkinson’s, and Huntington’s diseases [1]. However, amyloid pathologies include also other less know fatal neurodegenerative diseases such as transthyretin amyloidosis with polyneuropathy (ATTR-PN), or amyloidosis involving other organs such as the heart, as in the case of transthyretin amyloidosis with cardiomyopathy (ATTR-CM) or light chain amyloidosis (AL) [1].

The molecular mechanisms leading to organ failure in amyloid diseases are not completely understood. However, in the case of amyloid neurodegenerative diseases, it is known that the presence of oligomeric species produced in the amyloid aggregation cascade leads to neuron loss and progressive nervous system impairment. In the case of cardiac amyloid, although cardiomyocyte death is not commonly observed, cell-to-cell communication is significantly perturbed, which leads to heart failure [1]. Although, to date, there are no effective therapeutic solutions for Alzheimer’s, Parkinson’s, and most other amyloid diseases, significant progresses have been recently reached towards disease-modifying therapies in the case of ATTR and AL. In these cases, the most successful approaches are based on the stabilization of the native form of the amyloid precursor protein by pharmacological chaperones, or by dramatically decreasing the expression of the amyloid precursor protein using iRNA, antisense oligonucleotides (ASO) or chemotherapy. An alternative approach being pursued in vitro and in clinical trials is the removal of amyloid aggregates and fibrils from the affected tissues. This approach, depending on the particular technology being employed, is known by amyloid clearance, solubilization, fragmentation, breaking, untangling, disintegration, depolymerization, disaggregation, or disassembly. In the last two decades, this approach has gained particular attention and may be seen as an alternative promising approach to battle amyloidosis.

In the context of protein folding, molecular chaperones are proteins that prevent aggregation and promote the efficient folding of other proteins in the cell and thus contribute to maintain proteome homeostasis. Extending on this concept, research efforts have been concentrated on the identification of small molecules that might behave as chaperones, either by inhibiting protein aggregation, by stabilizing protein native states, by rescuing protein molecules trapped in misfolded states, or even by disassembling amyloid aggregates and fibrils, as shown by multiple examples in the literature [7,8,9,10,11,12]. These small molecules are commonly known as chemical chaperones or pharmacological chaperones and ideally should be able to disaggregate amyloid into non-toxic and off-pathway species. Some of these small molecules are particularly interesting as they can act as osmolytes, and they are mostly extracted from natural sources. The exact molecular mechanism on how chaperones exert disaggregation effects on preformed amyloid fibrils remains poorly understood. Understanding the interactions between chaperones and amyloid aggregates and fibrils rich in β-sheet structures is of paramount importance to fully understand the disaggregation mechanisms that lead to soluble and non-toxic oligomers and native monomers. Several computational and experimental studies have been conducted in order to identify the specific interactions that occur between amyloid fibrils and small molecule amyloid disruptors.

In this review, we focus on the role and therapeutic potential of molecular, chemical, and pharmacological chaperones in amyloid disassembly. In addition, we discuss specific disaggregation mechanisms according to the type of chaperone, in terms of their chemical structure. Understanding the specific interactions between chaperones and amyloid fibrils will be critical for developing new and more effective strategies to prevent and treat these highly debilitating and largely incurable proteinopathies known as amyloidoses.

## 2. Chaperones in Amyloid Disassembly

In amyloid disassembly, chaperones may be defined as proteins or compounds that are capable of solubilizing different protein aggregates and fibrils, such as amyloids implicated in misfolding diseases. Three main types of chaperones in amyloid disassembly have been identified, namely molecular chaperones, chemical chaperones, and pharmacological chaperones.

### 2.1. Molecular Chaperones in Amyloid Disassembly

Molecular chaperones are a family of proteins that plays an important role in protein homeostasis. Molecular chaperones can be classified into two main families, the ATP-independent chaperones and the ATP-dependent chaperones. In addition, the chaperone machinery can be grouped in distinct families, including ribosome-binding chaperones (e.g., Hsp40s and Hsp70s), chaperonins (e.g., Hsp60 and Hsp10), prefoldins, small heat shock proteins (Hsp), and tetratricopeptide (TPR)-domain containing co-chaperones.

Molecular chaperones function by repairing non-native protein conformations, for example, accelerating or delaying protein folding, preventing protein aggregation or mediating targeted disassembly, and mediating protein degradation via the ubiquitin/proteasome system (UPS) or via the autophagy/lysosome system (ALS) [13,14]. Chaperones can also interact with aberrant protein aggregates in the form of prefibrillar oligomers and amyloid fibrils with or without promoting their disassembly, and thus inhibit their toxicity commonly associated with neurodegenerative diseases [15,16]. However, the normal physiological levels of molecular chaperones eventually become functionally compromised by the accumulation of aberrant aggregates, leading to a decline in global protein homeostasis and to the dysregulation of multiple cellular pathways. This phenomenon increases with aging, which may be one of the main factors contributing to the late onset of several amyloidosis. The overexpression and induction of molecular chaperones have been shown to be protective in a wide range of animal models of amyloidosis [17]. Enhancing the disaggregation potential of molecular chaperones to revert protein aggregation is therefore an attractive strategy to delay the occurrence of protein aggregation-related pathologies.

#### 2.1.1. Hsp70 Chaperone Network

The biochemical characterization of this molecular chaperone system showed that it only contains a substrate-binding domain and an ATP-binding domain. To be effective as a molecular chaperone, Hsp70 requires a co-chaperone system that initiates its ATPase activity in order to release bound ADP and to start a continuous cycle of amyloid disaggregation. Hsp40, also known as J-domain proteins (JDP) belonging to class A (DNAJA1 and DNAJA2) and class B (DNAJB1 and DNAJB4), increase the low intrinsic ATPase activity of Hsp70. Hsp110 acts as a nucleotide exchange factor (NEF) and removes ADP after ATP hydrolysis, enabling a new Hsp70 interaction cycle with aberrant protein substrates [18]. Hsp70 recognizes a degenerate motif of about five residues enriched in hydrophobic amino acids, flanked by positively charged amino acids (statistically occurring every 30–40 residues in protein sequences) [19]. The surface exposure of this peptide motif in protein substrates allows for Hsp70 to promiscuously bind to different types of protein aggregates, including bacterial inclusion bodies formed upon overproduction of heterologous proteins, and protein aggregates and amyloid fibrils formed by misfolded proteins [20].

The disaggregase activity of this chaperone machinery is a delicate task and many mechanistic details of the Hsp110–Hsp70–Hsp40 system remain incompletely understood. Not only there are multiple components to consider, but also many members in each family of Hsp110, Hsp70, and Hsp40 proteins, with varying degrees of efficacy [21]. Efficient disaggregation mechanisms by Hsp70 were observed with several amyloid precursor proteins, namely α-synuclein [20,22,23], polyQ-expanded huntingtin [24], and tau [25,26].

Differential roles can be attributed to the J-domain protein classes in the disaggregation activity. Recently, studies on chaperone modulators have corroborated the idea that J-domain proteins of class A may preferentially act to prevent amyloid formation, acting predominantly on small aggregate species or monomers, while members of class B target fibrils and large oligomers for disaggregation [27,28].

Although the chaperone machinery has an effective disruptive action in amyloid fibrils, the activity may release monomeric and small oligomeric species, which in turn may induce new aggregation due to the resulting seeding-competent species [26]. In addition, amyloid fibril fragmentation may also enhance protein-misfolding cascades and exacerbate the formation of toxic oligomers [29].

Protein aggregation is part of the cellular response to an imbalanced protein homeostasis. Intense research interest to unravel the pathophysiological significance of this protein aggresome has unveiled the important role of molecular chaperones in proteostasis. Therefore, novel therapeutic strategies may aim at the function of chaperones in preventing aberrant protein misfolding and promoting the maintenance of cellular homeostasis.

#### 2.1.2. Hsp90

Heat shock protein 90 (Hsp90) is a highly abundant and ubiquitous molecular chaperone that plays an essential role in many cellular processes, including cell cycle control, cell survival, hormone, and other signaling pathways. In the last ten years, it has become a major therapeutic target for cancer, and viral and protozoan infections, but also in neurodegenerative disorders.

Hsp90 can disassemble preformed TDP-43 (transactive response DNA-binding protein 43) amyloid fibrils [30]. Furthermore, in association with nicotinamide mononucleotide adenylyl transferase 2 (NMNAT2), Hsp90 can function as a neuroprotective chaperone since it is capable of disaggregating and refolding previously aggregated species of tau [31].

Further studies are necessary to clarify the exact role of the molecular chaperone Hsp90 in disaggregating pathological amyloids and its role in protecting against toxicity in vivo.

#### 2.1.3. Tripartite Motif Proteins (TRIMs)

Tripartite motif proteins (TRIMs) constitute a large family of proteins with more than 80 members, all sharing the RBCC motif (N-terminal RING finger/B-box/coiled coil) [32]. The RING domain can act as an E3 ubiquitin or small ubiquitin-like modifier (SUMO) ligase to mark misfolded proteins for degradation [33]. TRIMs have been recently recognized as new ATP-independent human molecular chaperones capable of abolishing the aggregation of aberrant proteins and solubilizing preformed aggregates and amyloid fibrils. TRIM11 dissolves α-synuclein fibrils in vitro, converting them into soluble forms in a dose-dependent manner and suppressing α-synuclein toxicity, as seen in cell and mouse models of Parkinson’s disease (PD). Indeed, the intracranial delivery of adeno-associated viruses expressing TRIM11 suppressed the prion-like spread of α-synuclein pathology, neurodegeneration, and motor impairments in a PD mouse model [34]. The ability of TRIM11 to mitigate these phenotypes indicates that TRIM11 does not release toxic α-synuclein species [29]. TRIM11 also disassembled preformed fibrils of ataxin-1 with an expanded polyglutamine (polyQ) region (Atxn1 82Q) in a dose-dependent manner leading to the near-complete dissolution of the fibrils at a molar ratio of protein:chaperone of 1:5 or higher. In addition, TRIM11 also disaggregated fibrillar and amorphous aggregates of Atxn1 82Q [34], since Atxn1 82Q forms nuclear inclusions when overexpressed in mammalian cells [35]. TRIM11 not only prevented the further accumulation of Atxn1 82Q aggregates but also largely reduced pre-existing Atxn1 82Q inclusions, suggesting that TRIM11 can function as a molecular chaperone for misfolding-associated diseases in the cellular milieu [34].

Other TRIMs can also function as molecular chaperones in protein disassembly, including TRIM19 (also known as promyelocytic leukemia protein, PML) and TRIM21 [34,35]. In the nucleus, TRIM19 facilitates the degradation of an insoluble variant of ataxin-1 with an expanded polyQ repeat, which is linked to spinocerebellar ataxia [35]. TRIM19 also promotes the solubilization and degradation of other aggregated proteins, including polyQ-expanded huntingtin and the transactive response DNA-binding protein 43 (TDP-43) [35].

These findings suggest the need for, and the importance of, additional studies of TRIM proteins in order to better understand their action and their role in combating aberrant protein states and in protein disaggregation mechanisms.

#### 2.1.4. Lipocalin-Prostaglandin D Synthase (L-PGDS)

**Figure 2:**
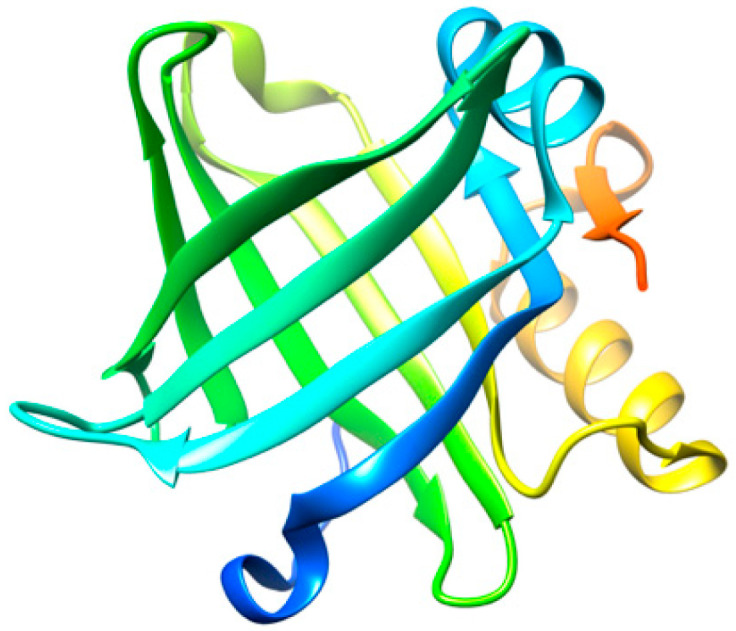
Three-dimensional structure representation of L-PGDS. Image produced with UCSF Chimera [6] using the crystallographic structure of L-PGDS wild-type in its apo form (PDB code: 4IMN).

Lipocalin-type prostaglandin D synthase (L-PGDS) (Figure 2) is one of the most abundant proteins in cerebrospinal fluid (CSF) with an estimated concentration of 15–30 μg/mL [36]. L-PGDS regulates multiple neurological processes and altered levels in the brain are related to the appearance of several pathological conditions [37,38]. In addition, L-PGDS exhibits a dual function as a molecular chaperone against Aβ aggregation [39], as well as being able to promote the disaggregation of preformed Aβ fibrils [40]. L-PGDS is known to bind to Aβ monomers and to Aβ fibrils with high affinity (K_d_ = 18–50 nM) through residues 25–28 in Aβ [39], which are also known to be involved in the mechanism of aggregation of the Aβ peptide [41].

The disaggregation efficiency of L-PGDS is relatively high, as L-PGDS exhibits disaggregase activity at a molar ratio of 1:0.1 (protein:chaperone) [40]. The concentration of L-PGDS in CSF under normal conditions would be sufficient to prevent the aggregation or promote the disaggregation of Aβ, since the concentration of Aβ40 (981 ± 409 pM) and Aβ42 (73.6 ± 41.8 pM) in AD patients is much lower than that of L-PGDS [42]. However, several studies have revealed that the level of L-PGDS in the CSF of AD patients is significantly reduced as compared to healthy controls [43,44].

#### 2.1.5. Transthyretin (TTR)

Transthyretin (TTR) is a globular homotetrameric protein mainly found in the plasma, cerebrospinal fluid (CSF), and the eye. TTR is known to transport thyroid hormones (thyroxine and triiodothyronine) and retinol (vitamin A) through binding to the retinol-binding protein (RBP). Tetramer dissociation and partial unfolding of TTR potentiates aggregation [45] and, therefore, the formation of amyloid fibrils, causing fatal amyloid diseases [46,47,48]. TTR amyloid deposits may be associated with wild-type TTR amyloidosis (ATTRwt), a relatively common cardiac disease of aging, but also to several TTR variants linked to hereditary cardiac and neurodegenerative amyloid diseases (ATTRv). A neuroprotective role against Alzheimer’s disease (AD) has also been assigned to TTR, acting as a molecular chaperone. There is evidence that TTR interacts not only with Aβ monomers [49,50,51], but also with Aβ aggregates [49]. The tetrameric form of TTR may redirect amyloid-β (Aβ) oligomeric nuclei into off-pathway species with a stoichiometry peptide:chaperone of 1:4 [52], as well as disrupt preformed Aβ amyloid fibrils into shorter fibrillar structures, smaller aggregates, and oligomers with a stoichiometry peptide:chaperone of 1:0.1 [53]. Significantly, TTR is able to prevent the cytotoxic effect of Aβ oligomeric species in neuronal cells [54,55]. Additionally, TTR monomers may also remodel soluble Aβ oligomers into amorphous aggregates at a molar ratio of peptide:chaperone of 1:0.5 [56]. Although the TTR concentration in CSF (90 to 360 nM) is higher than Aβ (Aβ40 981 ± 409 pM and Aβ42 73.6 ± 41.8 pM) [42,57,58], several studies report that TTR levels are diminished among AD patients when compared to controls [59], both in the CSF and plasma [60,61]. The cause for TTR reduction in AD patients is not yet fully understood.

**Figure 3:**
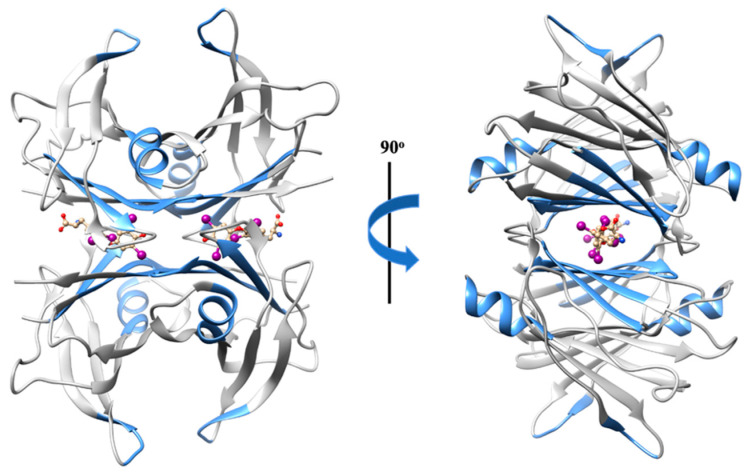
Three-dimensional structure representation of the native TTR homotetramer. The regions of TTR implicated in the interaction with Aβ are highlighted in blue. Image produced with UCSF Chimera [6] using the crystallographic structure of wild-type TTR in a complex with thyroxine (PDB code: 1ICT).

The Aβ binding domain engaged in the Aβ–TTR interaction involves residues 18 to 21 [50,62], the region already extensively described as highly amyloidogenic [41]. Hence, this may explain the disruptor effect exerted by the chaperone TTR against Aβ aggregates and fibrils. Interestingly, in turn, the region of TTR implicated in the peptide–chaperone complex appears to be non-specific. Several TTR regions may participate in the interaction (Figure 3), such as (1) residues 38 to 42 (β-strand C) of TTR monomers [63]; (2) β-strand A, which is solvent exposed in the monomeric conformation, but is sterically restricted in the tetrameric conformation [49]; (3) the α-helix, which is highly solvent exposed, both in monomers and tetramers; (4) residues 106 to 117 (β-strands G and H) [64]; and (5) residues in or around the thyroxine binding sites [50].

In AD, the main constituent of the extracellular amyloid deposits is the Aβ peptide in the form of senile plaques. Thus, the potential involvement of TTR in neuroprotection could open promising therapeutic approaches for AD.

#### 2.1.6. Cyclophilin 40 (CyP40)

**Figure 4:**
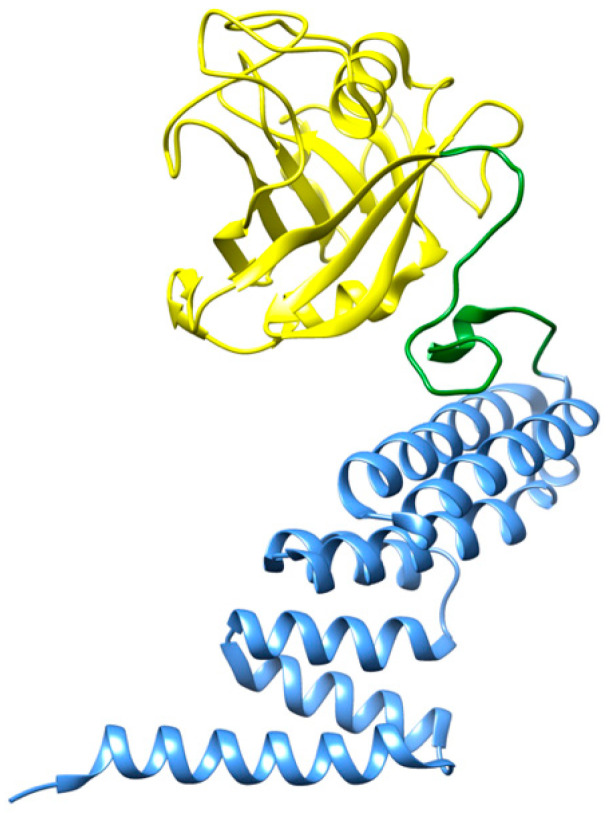
Three-dimensional structure representation of CyP40. The N-terminal domain of cyclophilin (residues 1–183) is shown in yellow, whereas the α-helical C-terminal TPR domain (residues 214–362) is shown in blue [66]. Image produced with UCSF Chimera [6] using the crystallographic structure of CyP40 (PDB code: 1IHG).

Cyclophilin 40 (CyP40) is a two-domain protein (Figure 4) that belongs to the family of peptidyl prolyl cis/trans isomerases (PPIase). Recent studies revealed that CyP40 is able to disaggregate proline-containing amyloids, as observed with α-synuclein fibrils and tau filaments [65]. The PPIase domain of CyP40 interacts with proline residues located at the β-turns of those amyloid fibrils and isomerizes these proline residues into an alternative configuration. This interaction disrupts the β-turn structure of the amyloid species and results in their disassembly. The disaggregating effects of CyP40 with tau filaments can be observed at molar ratios from 1:0.2 to 1:5 (peptide:chaperone). Conversely, CyP40 was not able to disaggregate preformed amyloid fibrils of amyloid-β (Aβ), since this peptide does not contain any proline residue along its polypeptide sequence [65].

Even though the disaggregation products of CyP40 were not explicitly characterized, the authors reported that overexpression of CyP40 can preserve the viability of neurons and rescue tau-induced cognitive decline in mouse models [65].

### 2.2. Chemical Chaperones in Amyloid Disassembly

**Figure 5:**
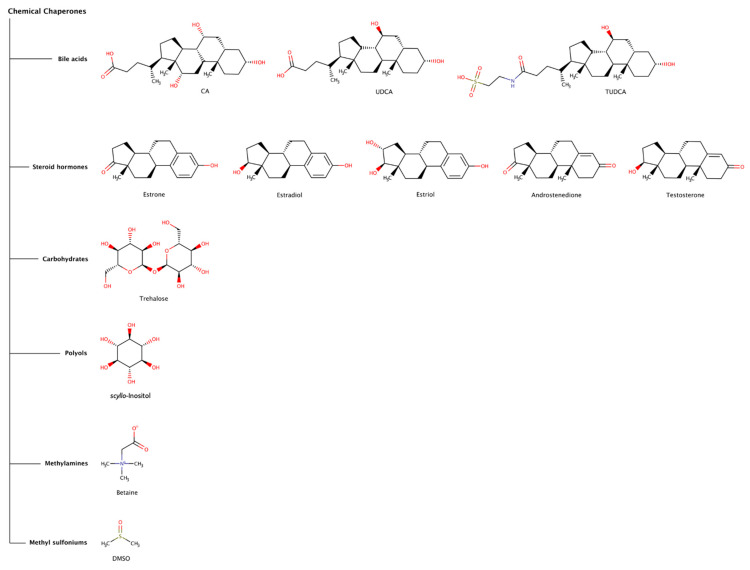
Chemical structures of chemical chaperones capable of disaggregating amyloid deposits.

Chemical chaperones refer to small molecules that can either stabilize the native state or destabilize or disaggregate misfolded or aggregated states of polypeptide chains. The most common types of chemical chaperones are hydrophobic compounds and osmolytes. Hydrophobic compounds include, for example, bile acids (e.g., deoxycholic acid (DCA), cholic acid (CA), ursodeoxycholic acid (UDCA), and tauroursodeoxycholic acid (TUDCA)), and steroid hormones (e.g., estrone, estradiol, estriol, androstenedione, and testosterone). Typical osmolytes are carbohydrates (e.g., trehalose), polyols (e.g., sorbitol, glycerin, and inositol), methylamines (e.g., betaine and trimethylamine N-oxide (TMAO)), methyl sulfoniums (e.g., dimethyl sulfoxide (DMSO) and dimethylsulfoniopropionate (DMSP)), among others [67,68]. Osmolytes are low-molecular-weight organic molecules that contribute to maintain the integrity of cells and biological tissues by regulating the osmotic pressure, but also viscosity, melting point and ionic strength of biological fluids. Naturally occurring osmolytes usually act as stabilizers of protein native structures. However, many other osmolytes come under the category of destabilizers (Figure 5), which may denature native states of proteins and promote solubilization of highly stable fibrillar protein conformations. Thus, it is understandable that, under stress conditions, cells may accumulate osmolytes in order to regulate protein homeostasis. Although most chemical chaperones usually have effect at high concentrations (sometimes in the millimolar and molar range), recently some chemical chaperones have been receiving increasing attention as potential therapeutic agents against proteinopathies, including intracellular amyloidoses.

#### 2.2.1. Bile Acids

Bile acids are a structurally related group of amphiphilic steroidal molecules derived from cholesterol that are widely known for their role as chemical detergents involved in the intestinal absorption and transport of fats and lipid-soluble nutrients [69]. Bile acids are readily bioavailable via oral, subcutaneous, or intravenous administration, can penetrate the blood–brain barrier (BBB), have low toxicity, and have been used as therapeutic agents directed to several clinical disorders, including amyloid diseases [70].

Cholic acid (CA) is the major primary bile acid synthesized from cholesterol in the hepatocytes. CA was found to prevent secondary nucleation and to drastically reduce the preformed fibrillar network in insulin fibrils, in a dose-dependent manner, suggesting a destabilizing/disaggregating effect [71].

The effect of tauroursodeoxycholic acid (TUDCA) has been studied in mice models of Alzheimer’s disease (AD). A treatment regimen with dietary supplementation with TUDCA significantly reduced amyloid plaques in the frontal cortex and hippocampus, decreased injury to neurons measured by the smaller loss of neuronal fibers surrounding amyloid plaques, and improved memory retention [72], as well as reduced hippocampal and prefrontal amyloid deposition [73]. TUDCA also gained clinical interest for the treatment of transthyretin amyloidosis (ATTR) since it has been referred to produce a decrease in the deposition of toxic pre-fibrillar TTR oligomers associated with ATTR amyloidosis in transgenic murine models [74,75]. The combination of TUDCA and the tetracycline antibiotic doxycycline, a pharmacological chaperone (see in more detail Section 2.3.3), has been demonstrated to synergistically reduce the amyloid fibril burden in a transgenic mouse model of ATTR, and to attenuate disease progression in clinical trials with a small number of patients [75,76]. However, the scope of the clinical trials was limited since the patients presented adverse side effects from doxycycline, namely gastrointestinal and skin events.

Ursodeoxycholic acid (UDCA) is a bile acid with an efficacy similar to TUDCA and has also been studied in combination with doxycycline for its fibril disruption effects in ATTR [77,78].

Although the evidence of clinical efficacy of bile acids is very limited, these findings may indicate that bile acids or similar molecules may be considered in therapeutic approaches against amyloid diseases.

#### 2.2.2. Steroid Hormones

Steroids are lipophilic hormones synthesized from cholesterol through a complex biosynthetic pathway in tissues and glands throughout the human body. Despite their shared molecular origin and basic structural similarities, there are four major classes of steroid hormones, namely corticoids (e.g., cortisol), estrogens (e.g., estradiol, estrone, and estriol), progestins (e.g., progesterone and aldosterone), and androgens (e.g., testosterone and androstenedione), which interact with high affinity with specific receptors to exert their biological effects. These hormones control diverse physiologic and cellular processes and affect almost all aspects of eukaryotic physiology, from sexual differentiation, growth, and reproduction to immunity, metabolism, and behavior [79]. Consequently, a clear and complete understanding of the basic mechanisms of steroid hormone action is of critical importance to health and disease.

The age-related loss of steroid hormones might be a risk factor for the development of several amyloidosis, such as ATTRwt amyloidosis, ATTRv with cardiomyopathy amyloidosis (ATTRv-CM), Alzheimer’s disease (AD), and Parkinson’s disease (PD). The reduction in estrogens in women and androgens in men may increase the vulnerability of the aging organs to progressive amyloid accumulation. In ATTRwt and ATTRv-CM, women showed signs of less severe cardiac impairment and myocardial involvement relative to men [80,81]. In AD and PD, a wide range of beneficial neural actions of steroid hormones have been described, which may contribute to neuroprotective roles against these amyloid diseases, some in a sex-specific manner [82,83], including protection from neuron death and clearance of amyloid content [84,85].

The disrupting effect of some steroid hormones on preformed amyloid fibrils of amyloid-β (Aβ) and α-synuclein was analyzed in experimental assays in vitro. Interestingly, the order of disaggregation activity of estriol, estradiol, estrone, androstenedione, and testosterone was quite similar in both amyloid systems, where estriol and estradiol were the hormones with the highest activity, and testosterone was the one with the lowest activity [84,85]. Curiously, the prevalence of AD and PD is recognized to be higher in women after the menopause [86]. In addition, the activities of estriol and estradiol on disassembly of α-synuclein fibrils are somewhat stronger than those observed for Aβ fibrils, since the molar ratio used for each study was 1:4 (Aβ:chaperone) and 1:1.4 (α-synuclein:chaperone), reaching the steroid hormones a maximum disaggregation effect of approximately 35% for Aβ fibrils and 50% for α-synuclein fibrils [84,85].

One possible explanation for the mechanism of action of these chemical chaperones is that steroid hormones may bind to the growing ends of preformed amyloid fibrils and increase the rate of depolymerization by destabilizing the conformation of the fibrillar structure at the fibril ends [84].

In terms of structure–activity relationships, estrone, estradiol, and estriol have one hydroxyl group in the C3 position of the A ring of the steroid hormone ring system, and exert significant anti-amyloidogenic effects, whereas androstenedione and testosterone do not (Figure 5). Estradiol, estriol, and testosterone have one hydroxyl group in the C17 position on the D ring (Figure 5), but their activities are different. The hydroxyl group in the C3 position might be essential for the destabilization activity and that in the C17 position might not. Additionally, the hydroxyl group in the C16 position on the D ring might justify the stronger activity of estriol relative to estradiol.

These findings might be correlated with the onset of some amyloidoses, since the reduction of estrogens with aging could accelerate Aβ plaques and Lewy bodies formation in the brain [82,83], and the reduction in androgens in men could play a role in accelerating TTR amyloid deposition in the heart [80,81]. In addition, estrogens may counteract nerve cell injury caused by Aβ [87]; enhance uptake of Aβ by microglia derived from the human cortex [88]; enhance dopaminergic function by increasing dopamine synthesis [89]; diminish oxidative stress, increase cerebral blood flow, and enhance cholinergic function and glucose transport into the brain [90]. Thus, estrogen could prevent or delay the development of neurodegenerative diseases by multiple mechanisms. These natural chemical chaperones may thus provide lessons to develop future therapeutic approaches directed to several human amyloidoses.

#### 2.2.3. Trehalose

The disaccharide trehalose has the ability to disassemble preformed amyloid fibrils of α-synuclein, amyloid-β (Aβ), and lysozyme [91,92,93] in vitro. A 2500-fold excess of trehalose over Aβ concentration had a greater disaggregation effect in Aβ40 than in Aβ42 [92]. For preformed amyloid fibrils of α-synuclein, a protein–chaperone stoichiometry of 1:700 resulted in small oligomers after long incubation periods [91]. However, a smaller excess of trehalose of only 20-fold over lysozyme concentration was sufficient to disassemble preformed lysozyme amyloid fibrils.

Trehalose appears to exert its amyloid disrupting effect by binding to aggregation prone regions in amyloid fibrils mostly through hydrogen bonds and eventually perturbing hydrophobic interactions. This leads to a reduction in the β-sheet content and results in disaggregation of amyloids [93]. Moreover, trehalose normally exerts a stabilizing effect on the native state of proteins.

Although trehalose is a non-toxic chemical chaperone with high solubility and oral bioavailability, the large molar excess of trehalose required for efficacious disaggregation activity may hinder its use in therapeutic applications.

#### 2.2.4. Scyllo-Inositol

Scyllo-Inositol, an endogenous myo-inositol isomer, has been shown to inhibit or reduce mutant huntingtin clumps in Huntington’s disease [94] and α-synuclein plaques in Parkinson’s disease [95]. In addition, treatment with scyllo-inositol has reversed cognitive deficits, reduced synaptic toxicity and lowered amyloid-β (Aβ) fibrils and plaques in a mouse model [96], and in clinical trials for Alzheimer’s disease (AD) [97]. Oral administration of scyllo-inositol (ELND005) at 10-fold excess over endogenous concentration levels (i.e., 1 mM) did not interfere with general physiological functions, such as phospholipid synthesis [98,99]. Nevertheless, the administration of high doses of scyllo-inositol (1000 or 2000 mg twice a day) were found to be deleterious in a randomized phase 2 clinical trial conducted in 353 patients with mild or moderate Alzheimer’s disease (AD). Although the high doses were discontinued due to significant incidence of adverse reactions in patients, the trial continued with only a lower dose (250 mg twice a day) but with no significant cognitive or functional improvement [97]. Even if the clinical trial helped to establish the safety profile of scyllo-inositol, the removal of the higher dose groups reduced the significance of the study to establish efficacy [100].

#### 2.2.5. Betaine

The osmolyte betaine has a dual role at two different molar concentrations, acting as a disaggregator at high molarity, but also as a stabilizer at low molarity [101]. This osmolyte has been shown to disaggregate amyloid fibrils derived from the fusion protein glutathione-S-transferase-green fluorescent protein (GST-GFP) in a dose-dependent manner (>10 M) into soluble oligomers. In addition, betaine was able to partially recover the secondary structure of the protein; however, it could not restore the tertiary structure of GST-GFP. The disassembled products could then be targeted by chaperones or proteasome complexes, which could either recycle the functional protein or degrade and clear the disaggregation products that were unable to regain function [101]. According to the authors, high concentrations of betaine can penetrate cells, activating other chaperones responsible for the disaggregation of amyloid aggregates [102].

#### 2.2.6. Dimethyl Sulfoxide (DMSO)

In recent decades, dimethyl sulfoxide (DMSO) has been widely used in preclinical and clinical research. Pharmacological effects of DMSO have been reported, and some of its biological activities have shown to be beneficial against diverse pathologies [103]. However, DMSO doses of 10% or higher are toxic in vivo [104]. Intriguingly, particularly low DMSO concentrations appear to profoundly influence neural network activities [105]. Moreover, chronic DMSO treatment has been shown to attenuate spatial memory deficits induced by ischemia [106], suggesting that prolonged DMSO administration has protective actions for the central nervous system (CNS) under pathological conditions. In misfolding-related disorders, a destabilizing effect of DMSO was observed on preformed amyloid fibrils of β2-microglobulin and insulin in vitro. At a concentration of 80% (v/v), DMSO completely dissolved preformed β2-microglobulin fibrillar structures at low pH [107], while at 90% (w/w) DMSO fully unfolded highly ordered structures of insulin into random coil deposits [108].

The disrupting effect of DMSO can be attributed to the hydrogen-bonding character of its oxygen atom that may interact with some amino acid side chains located at the surface of the cross-β motif of amyloid fibrils [107] but also with the peptide bonds, followed by preferential solvation of hydrophobic side chains by the DMSO methyl groups [109].

### 2.3. Pharmacological Chaperones in Amyloid Disassembly

Pharmacological chaperones, or pharmacoperones, are attractive for potential therapeutic applications because they are active at relatively low concentrations in comparison to chemical chaperones, but they have effects on par with molecular chaperones. This class of chaperones entails low-molecular-weight molecules, which exert their action by binding specifically to target proteins. They may inhibit aggregation and fibrillation by stabilizing soluble native protein conformations and avoiding protein misfolding. However, they may also lead to the formation of non-toxic and off-pathway oligomers or they may destabilize aggregated states.

Tafamidis, the archetypal pharmacoperone stabilizer, is a relatively recent medicinal drug used in the treatment of transthyretin amyloidosis (ATTR). Tafamidis acts by stabilizing the homotetrameric native form of the protein transthyretin and some of its more amyloidogenic variants (naturally occurring single site mutations) by binding at the two thyroxine-binding sites of the protein [110]. ATTR has multiple clinical manifestations, involving different organs, but the TTR amyloid cascade is always initiated by tetramer dissociation to low conformational stability monomers that partially unfold and aggregate into amyloid fibrils [45,47,111,112]. Thus, the stabilization of the native form of the protein decreases amyloid formation.

In the case of amyloid destabilizing pharmacological chaperones, there are obvious practical limitations to their use to treat amyloidoses—the relatively high concentration of the biological target, especially in the case of amyloid fibrils, and the localization of the biological target. To target intracellular amyloid, the compounds must be able to enter the cells through passive diffusion or receptor-mediated transport at sufficiently high concentrations to be efficacious. Moreover, if the target compartment is the central nervous system (CNS), the pharmacoperone must permeate protective barriers such as the blood–brain barrier (BBB) or the blood–cerebral spinal fluid barrier (BCSFB). The concentration of the pharmacological chaperone required to revert the amyloid state might be high enough to become toxic or produce undesired side effects. Pharmacological chaperones with a short half-life might require continuous infusion of the compound or slow-release formulations, complicating drug development or drug administration. Despite these potential challenges, several in vitro and in vivo assays and clinical trials suggest that pharmacological chaperones offer an exciting therapeutic strategy to treat intra- and extracellular amyloidosis.

#### 2.3.1. Amino-Acid Derivatives

**Figure 6:**
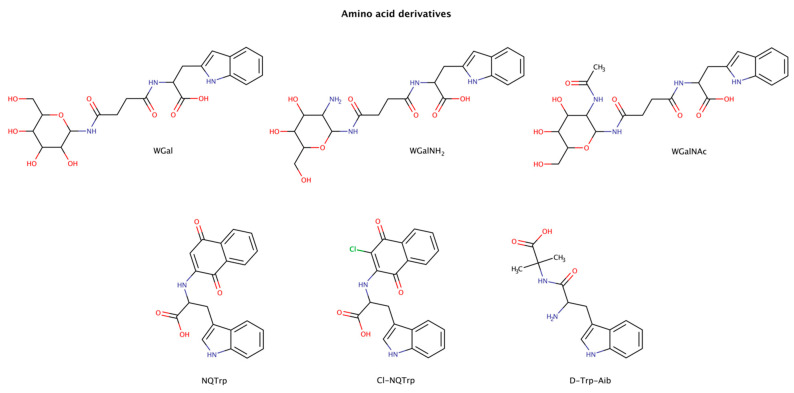
Chemical structures of tryptophan derivatives with disruptive effects on preformed amyloid structures.

Combining the aromatic elements of amino acids with other small molecules has been an attempted strategy for amyloid inhibition and disassembly (Figure 6). The amino acid tryptophan was shown to be highly effective in intercalating into amyloid fibrils of various proteins and in inhibiting their aggregation [113,114,115,116,117,118]. The combination of tryptophan with naphthoquinones was shown to be active in the prevention of aggregation in various amyloid systems, such as amyloid-β (Aβ), islet amyloid polypeptide (IAPP), tau, and α-synuclein, both in vitro and in vivo [113,114,115,118,119]. Moreover, some naphthoquinone-tryptophan molecules, NQTrp and Cl-NQTrp, could also disaggregate preformed amyloid fibrils of full-length tau protein and the hexapeptide sequence ^306^VQIVYK^311^ (PHF6) in a dose-dependent manner [113]. The maximum disassembly (reduction in approximately 40% amyloid content) was achieved with 1:5 molar ratio (protein:chaperone), which also led to an evident decrease in the β-sheet content, a significant reduction in fibril density, and lack of elongated fibrillar structures. Furthermore, the disassembled intermediate products were less toxic than the initial oligomers [113].

A major limitation in the therapeutic use of these amphiphilic or hybrid molecules is their poor solubility in aqueous environments. To overcome this limitation, attaching a more hydrophilic moiety may increase the solubility and enhance the overall therapeutic efficacy of the molecule [120]. Functionalization with glycans has been recognized to increase solubility, half-life and specificity of certain drugs in vivo [121]. Tryptophan-galactosylamines molecules (WGal, WGalNH_2_, and WGalNAc) have been confirmed to disrupt preformed amyloid fibrils of Aβ and IAPP with a maximum activity at a molar ratio of 1:5 (protein:chaperone) [122].

The designed molecule D-Trp-Aib (D-tryptophan-α aminoisobutyric acid) combines the aromatic amino acid tryptophan with the β-sheet breaker α-aminoisobutyric acid [123]. D-Trp-Aib was tested using a 20-fold excess to IAPP or calcitonin, and a 30-fold excess to α-synuclein preformed amyloid fibrils, and was able to disaggregate the fibrillar structures. The authors hypothesized that the use of β-breaker elements combined with aromatic moieties may present a promising general approach for the development of amyloid inhibitors. 

In order to understand the interactions involved between fibrillar structures and pharmacological chaperones, molecular dynamics (MD) simulations were performed in an amyloid fibril system and tryptophan hybrid molecules [113]. The MD analysis suggested a plausible mechanism of disassembly either in the oligomer or fibril conformation, where tryptophan hybrid molecules interact with β-sheet-rich structures through hydrophobic effects via π–π stacking interactions in combination with the formation of hydrogen bonds, with crucial residues responsible for β-sheet formation [113].

Aromatic residues, especially phenylalanine and tryptophan, are relatively abundant in amyloidogenic sequences [124], with tryptophan being the amino acid residue with highest amyloidogenic potential [116]. Thus, the use of chemical chaperones with an aromatic moiety may facilitate the molecular recognition of amyloidogenic sequences improving the directionality and orientation necessary for the interaction and disaggregation of these highly ordered fibrillar species.

#### 2.3.2. Anthraquinone Derivatives

**Figure 7:**
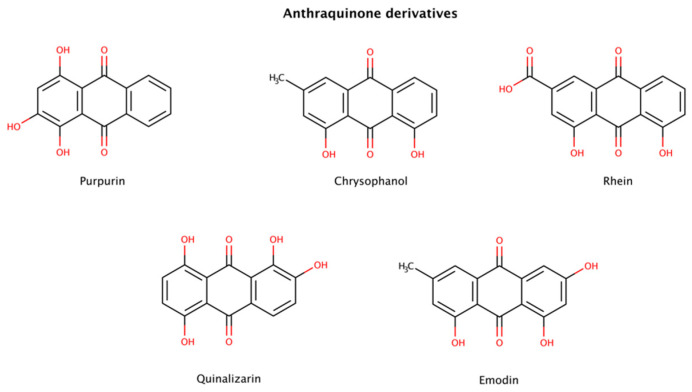
Chemical structures of anthraquinone derivatives with disruptive effects on preformed amyloid fibrils.

Among several compounds identified as amyloid disruptors, anthraquinones (Figure 7), planar and tricyclic compounds, have recently attracted attention. On the one hand, natural anthraquinone-rich plants have been used as traditional Chinese medicine for a long time to treat several medical conditions. On the other hand, modern scientific research has also reported that anthraquinone derivatives have a variety of pharmacological activities including antioxidant, anti-inflammatory, antibacterial, and anticancer properties [125,126]. Previous studies reported that certain anthraquinones could effectively disrupt preformed amyloid fibrils. For example, purpurin, a naturally occurring anthraquinone dye pigment, had a dose-dependent disruptor effect on preformed aggregates from the hexapeptide fragment ^306^VQIVYK^311^ (termed PHF6) derived from tau. Purpurin disassembled up to 75% and 50% of preformed PHF6 fibrils at 5-fold and 2-fold molar excess, respectively, over a period of only 1 h. A molecular dynamics (MD) study showed that purpurin interacts with preformed PHF6 fibrils (PDB code: 2ON9) essentially via hydrogen bonds and hydrophobic contacts with crucial residues responsible for the β-sheet arrangement, therefore facilitating amyloid disassembly. Using an animal model and a cell model overexpressing tau, purpurin was also able to alleviate Alzheimer’s disease (AD)-like symptoms and reduce the accumulation of total tau, respectively. In addition, it was observed that purpurin may cross the human blood–brain barrier (BBB) efficiently [127].

Emodin and quinalizarin were also tested with mature fibrils of various constructs of tau. The DC50 (compound concentration at which 50% of the target protein is disassembled) obtained for emodin were 2.8 μM (K19), 2.0 μM (K18), 3.9 μM (K18ΔK280), 7.0 μM (hTau23) and > 60 μM (hTau24), whereas for quinalizarin were 2.2 μM (K19), 7.3 μM (K18), 0.7 μM (K18ΔK280), > 60 μM (hTau23) and 39.2 μM (hTau24), using a total protein concentration of 10 μM [128].

Low extent disaggregation at the early stages of aggregation was also detected in chrysophanol- and rhein-treated insulin samples [129].

Taken together, these results indicate that anthraquinones seem to be an attractive lead compound for drug development in amyloidosis.

#### 2.3.3. Benzophenone Derivatives

**Figure 8:**
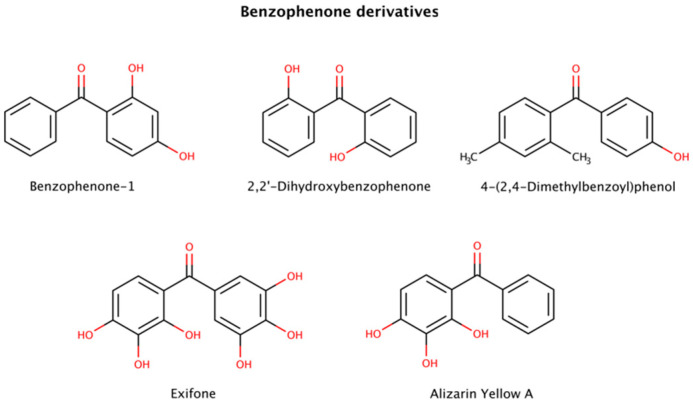
Chemical structures of benzophenone derivatives with disruptive action on preformed amyloid species.

Benzophenone (diphenyl ketone) derivatives (benzophenones) are widely used to protect against UV light in sunglasses, food packaging, laundry and cleaning products, inks, and in personal care products, among others (Figure 8). However, benzophenone is considered a mutagen, carcinogen, and endocrine disruptor and its presence in food products or food packaging is banned in the United States.

This class of molecules exhibits a range of biological activities, including antifungal, anti-HIV, antimicrobial, antioxidant, antiviral, antiparasitic, and cytotoxic [130]. Furthermore, benzophenones have been implicated in amyloid clearance by disaggregating preformed aggregates and amyloid fibrils of various amyloidogenic proteins.

Three benzophenone derivatives, benzophenone-1 (2,4-dihydroxybenzophenone), 2,2′-dihydroxybenophenone, and 4-(2,4-dimethylbenzoyl)phenol, showed an active disruptor effect at 8-fold molar excess relative to amyloid-β (Aβ) peptide [131]. Further assays involving exifone with preformed Aβ fibrillar species displayed a weak disaggregation effect at equimolar ratios [132].

Benzophenone derivatives were also tested with several constructs of mature tau fibrils. Alizarin Yellow A in disaggregation assays exhibited 3.8 μM (K19), 20.8 μM (K18), 3.1 μM (K18ΔK280), > 60 μM (hTau23), and 10.8 μM (hTau24) DC50s (compound concentration at which 50% of target protein is disaggregated) [128].

Polyphenols are suspected to target π-stacking interactions in aggregated species of amyloidogenic polypeptides [124]. Thus, it is possible that benzophenones initiate the amyloid remodeling process by disrupting π-stacking intermolecular contacts between oligomers and growing fibrils [131].

#### 2.3.4. Catechin Derivatives

Catechin is a plant metabolite with antioxidant properties, present in human dietary sources such as tea, cacao, grapes and other fruits. Catechins are however extensively metabolized upon uptake from the gastrointestinal tract and by the gut microbiome. The effects of catechins are wide ranging, with known beneficial activities such as antioxidant, antimicrobial, antiviral, anti-inflammatory, anti-allergenic, anticancer, and anti-amyloidogenic [133].

**Figure 9:**
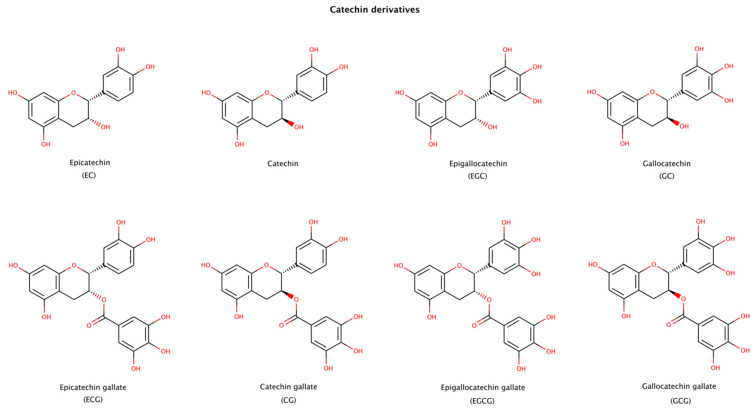
Chemical structures of catechin derivatives reported to disassemble amyloid fibrillar structures.

Epigallocatechin gallate (EGCG), a natural catechin extracted from the tea plant *Camellia sinensis*, is one of the most studied molecules in amyloid aggregation and disaggregation assays, since it both inhibits fibril formation and disaggregates fibrils of numerous amyloid precursor proteins. Catechins presented in Figure 9 have been reported to disaggregate preformed amyloid fibrils. The products formed after disaggregation can be either small or large amorphous aggregates, oligomers, partially unfold oligomers, or unfold monomers [134]. Among the catechins, EGCG usually displays the largest effect on amyloid disassembly [135,136,137]. EGCG contains an extra gallic acid ester group compared to catechin, EC, EGC, and GC, and an extra hydroxyl group compared to ECG and CG (Figure 9). These additional groups seem to provide a strong disruptive effect to EGCG. The stereoisomer of EGCG, GCG, is also an effective disruptor, although to a lesser extent than EGCG [136,138].

In order to better understand the interactions between catechins and amyloid fibrils, several molecular dynamics (MD) simulations have been recently performed with EGCG. These studies showed that EGCG can disrupt Aβ42 protofibrils rich in β-sheet content (PDB codes: 5OQV, 2BEG, 2NAO, and 2MXU) through hydrophobic, π–π stacking, and hydrogen-bonding interactions. EGCG also disrupted salt bridges through hydrogen-bonding interactions and cation−π interactions between its gallic acid ester moiety and key residues in the amyloid structure. These studies also revealed that EGCG mostly interacted with residues whose side chains point outwards from the surface of the protofibril [138,139,140]. In a study with a newly solved α-synuclein fibril composed of two intertwined protofibrils (PDB code: 6CU7), EGCG was shown to reduce the structural stability of the fibril through disruption of β-sheets in the N- and C-terminal regions, destruction of one salt-bridge, and dissociation of the inter-protofibril interface [141]. Another study showed that EGCG disaggregates tau fibrils by wedging into a cleft that is at the interface of two protofilaments of the paired helical filament, and by causing charge repulsions between tau layers of the fibril [142].

Isothermal titration calorimetry (ITC) assays were conducted in order to obtain the dissociation constant (K_d_) of EGCG and amyloid fibrils of tau and γ-synuclein. The results showed values of K_d_ in the μM and mM range, respectively [142,143].

This class of small molecules is particularly interesting as they are mostly extracted from natural sources (plants) and have low cytotoxicity. Preliminary clinical data in ATTR patients with cardiomyopathy amyloidosis (ATTRwt and ATTRv-CM) consuming 500–700 mg of EGCG daily for 1 year showed significant reductions in amyloid content in the myocardium [144,145]. However, another study in ATTR patients with cardiac involvement treated with EGCG (675 mg daily dose) for a minimum of 9 months showed no difference in survival estimates between EGCG-treated and control groups, although EGCG was well tolerated, without major safety concerns [146].

Various epidemiological studies have provided some evidence indicating that green tea consumption is associated with a reduced risk of age-related cognitive decline and AD [147], and a lower prevalence of cognitive impairment in PD [148]. Nevertheless, additional evidence derived from placebo-controlled, well-designed clinical assays are highly needed and recommended in AD and PD patients.

Despite their potent in vitro anti-amyloid activity, catechins have poor drug-like properties, such as limited bioavailability, fast metabolism and elimination, and lack of specificity. Catechins are known to interact with numerous protein targets due to their promiscuous activity [149]. Despite the limitations of some catechins as clinical agents to treat chronic diseases as amyloidosis, understanding how these molecules interact with cross-β structures is of paramount importance to develop new insights for the discovery of potent and effective pharmacological chaperones with drug-like properties.

#### 2.3.5. Anthracycline and Tetracycline Derivatives

**Figure 10:**
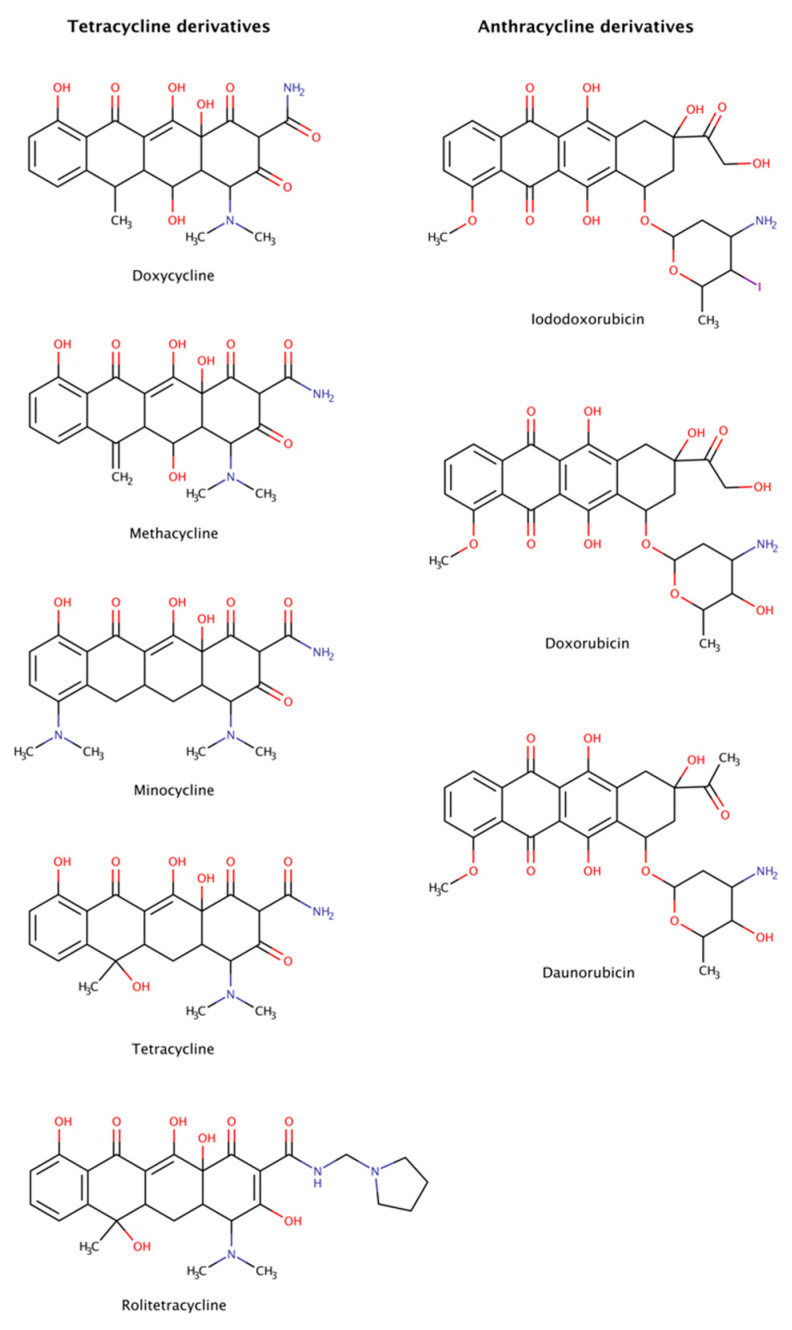
Chemical structures of tetracycline and anthracycline derivatives able to disassemble preformed amyloid structures.

Anthracyclines and tetracyclines are two classes of medical drugs characterized by a planar tetracyclic chromophore. Anthracyclines have an anthraquinone motif containing an amino sugar group, and tetracyclines contain a naphthacene carboxamide ring system (Figure 10). The members of both families present an ample range of biological activity. On the one hand, anthracyclines are effective as antibacterial, immunosuppressants, antiparasitic, and antitumor agents. Two anticancer anthracyclines, doxorubicin and daunorubicin, are used clinically and are among the most efficient antitumor drugs [150,151]. On the other hand, tetracyclines are commonly used against bacteria, but also present biological actions that affect inflammation, acne, proteolysis, angiogenesis, apoptosis, metal chelation, ionophoresis, and bone metabolism. 

Anthracyclines and tetracyclines have also been tested as amyloid disruptors [152,153]. Numerous in vitro and in vivo assays, as well as clinical trials, have been performed. 

The amyloid systems tested to date with tetracyclines were amyloid-β (Aβ) [154], α-synuclein [155,156], transthyretin (TTR) [46,157,158], β2-microglobulin (β2m) [159], islet amyloid polypeptide (IAPP) [160,161], immunoglobulin (Ig) light chain (LC) [162], prion protein (PrP) [163], and polyQ [164]. These experiments revealed that tetracyclines are capable of not only completely disassemble amyloid fibrils, but also of blocking assembly into amyloid fibrils, generating non-toxic species after disaggregation, preventing neuronal death and, in some cases, producing clinical benefits in patients affected by amyloidosis. However, these drugs are often poorly tolerated, producing significant side effects such as diminished production of blood cells, gastrointestinal problems, and increased risk of infection [165].

Anthracyclines were tested against amyloid systems like TTR [157,166], tau [128], Ig LC [167], amyloid A, Aβ, and β2m [168]. Anthracyclines interacted with amyloid fibrils of various compositions facilitating their rapid dissociation and clearance and the resulting products were not associated with cytotoxicity.

Despite the dissimilarities in sequence, structure, and function of all the protein precursors studied, amyloid fibrils share many common morphological features forming long, unbranched filaments with a cross-β sheet conformation [1]. The data suggest the existence of a universal mechanism of action of tetracyclines and anthracyclines against amyloids, targeting certain structural features of amyloid fibrils, as the cross-β-sheet quaternary structure, which could make these drugs potential anti-amyloidogenic pharmacological chaperones for all types of amyloidosis. Nevertheless, the clinical utility of anthracyclines is limited by its dose-dependent cardiotoxicity that adversely affects 10–30% of patients [169] and leads to devastating adverse effects resulting in poor quality of life, morbidity, and even premature mortality [170].

Molecular dynamics (MD) simulations were run in an attempt to explain the molecular mechanisms of destabilization of preformed amyloid fibrils by tetracyclines and anthracyclines [171]. The results showed that doxycycline employed at a 1:1 molar ratio tightly binds an exposed hydrophobic region of Aβ42 amyloid fibrils (PDB code: 2MXU), partly leading to the destabilization of the fibrillar structure. In addition, iododoxorubicin or IDOX molecules formed stable interactions with two hydrophobic pockets of the fibrillar structure (PDB code: 2MXU) also at a 1:1 molar ratio with respect to Aβ peptides. These contacts were stabilized by the hydrophobic interactions between the rings of IDOX and the side chains of the Aβ fibrils. A third binding region corresponded to a solvent-exposed surface of a larger hydrophobic core. These drugs strongly interacted with amyloid fibrillar structures, binding to their surface in exposed hydrophobic regions and, therefore, showed potential for destabilizing amyloid fibrils [171].

Tetracyclines and anthracyclines have advantages over other newly proposed anti-amyloidogenic drugs, thanks to their well-known and characterized pharmacokinetics and pharmacological properties [172,173]. However, anthracyclines cross only poorly the blood–brain barrier (BBB) and display significant cardiac toxicity, and tetracyclines constitute a group of antibiotics with adverse effects associated with dermatologic and gastrointestinal symptoms [78], but with lower toxicity and acceptable permeation of the BBB.

All this information indicates that there is room for improving the therapeutic index of these drugs towards the development of amyloid destabilizers and new treatment options.

#### 2.3.6. Non-Steroidal Anti-Inflammatory Drugs (NSAIDs)

**Figure 11:**
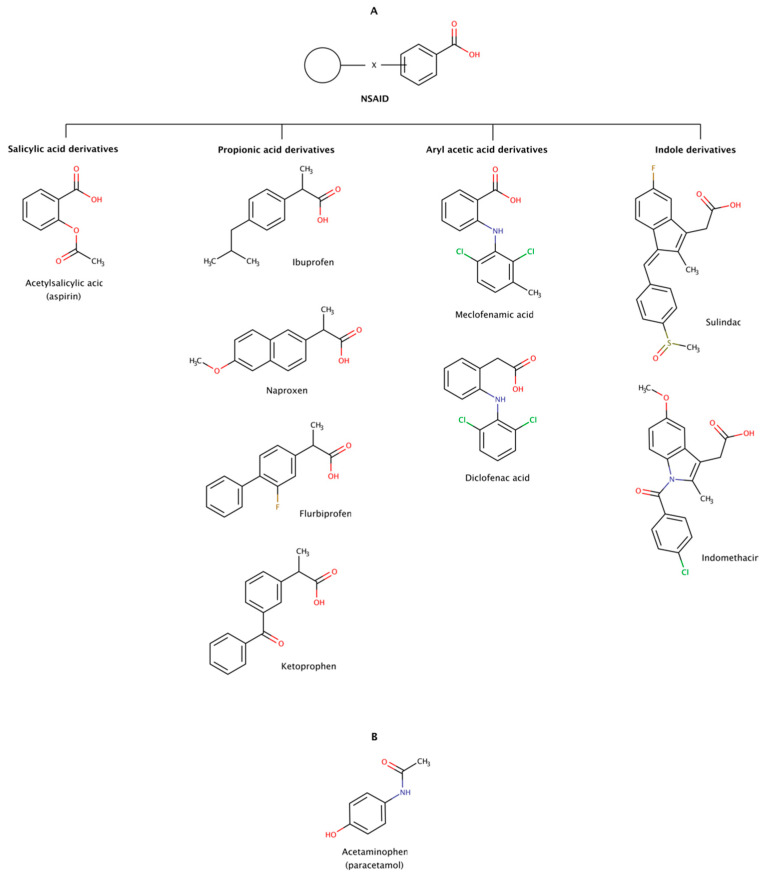
Chemical structures of NSAIDs (A) and paracetamol (B) with the ability to disaggregate amyloid fibrils in vitro.

Non-steroidal anti-inflammatory drugs (NSAIDs) are a heterogeneous group of therapeutic molecules used as antipyretic, anti-inflammatory, and analgesic agents. These effects make NSAIDs useful for the symptomatic treatment of several pathological conditions, such as arthritis, fever, and pain. This class of drugs includes ibuprofen and aspirin to reduce pain, fever, or inflammation; and naproxen and diclofenac usually used to relieve pain. Thus, every day, there are millions of patients taking NSAIDs, making this class of drugs one of the most widely used worldwide. Figure 11 shows examples of several classes of NSAIDs and the general structure of a typical NSAID, with an acidic functional group (carboxylic acid) attached to a planar aromatic ring. 

More than 90% of the NSAIDs are highly bound to serum proteins. These drugs normally exhibit considerably good bioavailability in monogastric organisms upon oral, subcutaneous, and intramuscular administration due to their low molecular weight and moderate to high degree of lipid solubility. These physico-chemical properties allow them to permeate into the brain through the blood–brain barrier [174].

In addition to their traditional uses in medicine, limited clinical trials have indicated that NSAIDs may be directed to some amyloidoses, either by inhibiting protein aggregation or by disassembling preformed amyloid fibrils. For example, diclofenac, diflunisal, and flufenamic acid and their derivatives are known to stabilize in vitro the native conformation of several transthyretin (TTR) amyloidogenic variants and inhibit amyloid fibril formation [175]. Diflunisal has also been administrated in TTR amyloidosis (ATTR) patients and showed some efficacy on arresting the progression of amyloidosis with polyneuropathy (ATTRv-PN) and amyloidosis with cardiomyopathy (ATTRv-CM and ATTRwt) [176,177,178]. Despite its potential gastrointestinal, renal, cardiac, and hematological adverse effects, the use of diflunisal was reported to be safe by several single-center clinical trials [176,177,179].

The disaggregation effect of nine NSAIDs (Figure 11) has been observed in preformed amyloid fibrils of amyloid-β (Aβ) and α-synuclein, the precursor proteins responsible for Alzheimer’s disease (AD) and Parkinson’s disease (PD), respectively [180,181]. The results showed disrupting effect in preformed amyloid fibrils of Aβ40 and Aβ42. The disrupting activity of these pharmacological chaperones determined at a molar ratio of 1:2 (protein:chaperone) was in the following order: ibuprofen ≈ sulindac sulfide (a metabolite of sulindac) ≥ meclofenamic acid > aspirin ≈ ketoprofen ≥ flurbiprofen ≈ diclofenac acid > naproxen ≈ indomethacin. The same authors observed a similar disaggregation effect of NSAIDs on preformed amyloid fibrils of α-synuclein at a molar ratio of 1:0.7 (protein:chaperone): ibuprofen ≈ aspirin ≈ acetaminophen ≈ meclofenamic acid ≈ sulindac sulfide > ketoprofen ≈ flurbiprofen ≈ diclofenac acid > naproxen ≈ indomethacin. The disaggregation effect on both cases showed a markedly decreased of fibrils and occasionally formation of small amorphous aggregates in vitro. Ibuprofen, sulindac sulfide, aspririn, meclofenamic acid, and ketoprofen were the most active NSAIDs in disrupting preformed amyloid fibrils in vitro in both studies, i.e., in Aβ and α-synuclein [180,181].

Molecular docking was performed to identify potential sites of interaction of NSAIDs with fibrils of Aβ. A dataset of 23 NSAIDs plus acetaminophen was docked into a fibrillar structure of Aβ (PDB code: 2BEG). All docked compounds were ranked according to their binding energy. The in silico results showed variable affinities with the Aβ fibril, curiously occupying the same binding region of the fibril structure localized between two β-strands on an hydrophobic region encompassing residues 17–21 (LVFFA). This pentapeptide region was also previously implicated in the mechanism of amyloid fibril formation by Aβ [41]. Leu17, Val18, and Phe19 participate in van der Waals interactions with the chaperone molecule, and Gly37 and Val39 backbone atoms contribute with hydrogen bonds. The NSAIDs with lowest binding energy were ranked as follows: sulindac (−12.44 kcal/mol) > meloxicam (−12.24 kcal/mol) > oxaprozin (−12.04 kcal/mol) > celecoxib (−11.91 kcal/mol) > nimesulide (−11.58 kcal/mol) > mefenamic acid (−10.81 kcal/mol) > diflunisal (−10.28 kcal/mol) > acetaminophen (7.45 kcal/mol) [182].

Although these findings seem to indicate that NSAIDs could be relevant therapeutic agents against amyloid diseases, it is today well known that long-term use of NSAIDs leads to gastro-intestinal and cardiac adverse effects. Moreover, incidents of cognitive impairment in aspirin users and risk of dementia in aging subjects highlights safety concerns and potential neurotoxic effects in AD patients [183,184,185,186].

#### 2.3.7. Porphyrin and Phthalocyanine Derivatives

**Figure 12:**
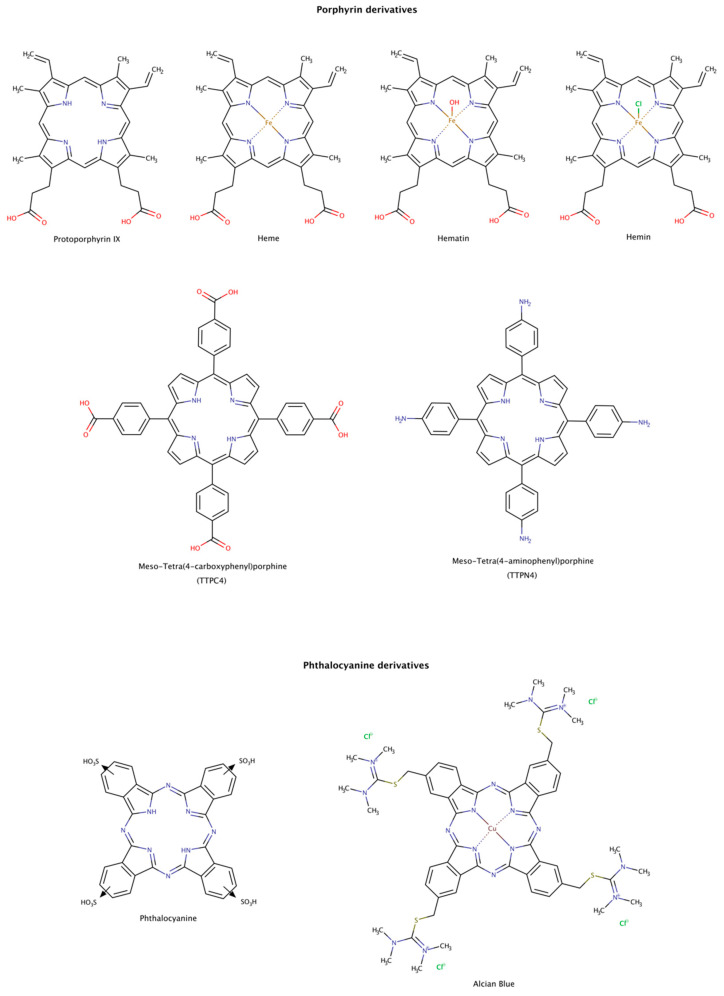
Chemical structures of porphyrin and phthalocyanine derivatives known to disassemble preformed amyloid fibrils.

From the structural comparison of the multitude of compounds able to disaggregate preformed amyloids, the most active compounds do contain aromatic cores [187]. Their action may be mediated by stacking interactions between their aromatic moieties and the side chains of aromatic amino acids in the target proteins [138,188,189]. Among the aromatic macrocycles, the heme-like complexes such as porphyrins and phthalocyanines have been widely studied as modulators of amyloid formation as inhibitors [187,190] and as disruptors (Figure 12) [191,192,193,194,195].

Hemin, a ferric iron-containing protoporphyrin IX, induced the dissociation of preformed lysozyme amyloid fibrils mainly into monomeric species. The dissociation was found to be concentration-dependent and reached a maximum at protein:chaperone molar ratio of only 1:2. The dissociated monomers have characteristics of a partially folded intermediate state of the native protein [191].

Heme and protoporphyrin have been tested in assays with preformed amyloid-β (Aβ) fibrils. The results showed that both compounds caused a conformational transition to unordered conformations, suggesting a dissociation mechanism of the fibril into Aβ monomeric species. Heme effectively disaggregated amyloid fibrils at a molar ratio protein:chaperone of 1:0.4, whereas protoporphyrin showed weaker effects at the same molar ratio. This observation may be explained by the interaction between the porphyrin ring and Phe19 at the C-terminal hydrophobic region of Aβ. This interaction facilitates the binding of the complex and perturbs the inter-strand aromatic interaction between Phe residues, which may facilitate amyloid disassembly. In addition, the iron center also influences the binding affinity of the heme to His residues in the N-terminal hydrophilic region of Aβ, as well as the rate and extension of disaggregation. The synergy of the two actions makes the heme an effective amyloid disruptor agent [192].

Preformed tau amyloid fibrils were incubated with hematin and phthalocyanine. Once again, these pharmacological chaperones led to a concentration-dependent increase in tau subunits, suggestive of the disassembly of tau filaments [193]. The disassembly of tau filaments was also observed in the presence of Alcian Blue [194].

The disassembly effect of tetraphenylporphyrins on mature insulin fibrils has also been investigated. To determine the disruptor effect of TPPC4 and TPPN4 (Figure 12), preformed insulin fibrils were incubated with these compounds at equimolar ratio of protein-compound (1:1). After incubation for 6 days, the compounds showed a weak disruptor effect [195].

Although porphyrin and phthalocyanine derivatives are not oral bioavailable and may present phototoxicity, their use could eventually be explored against some amyloid diseases.

#### 2.3.8. Surfactants

**Figure 13:**
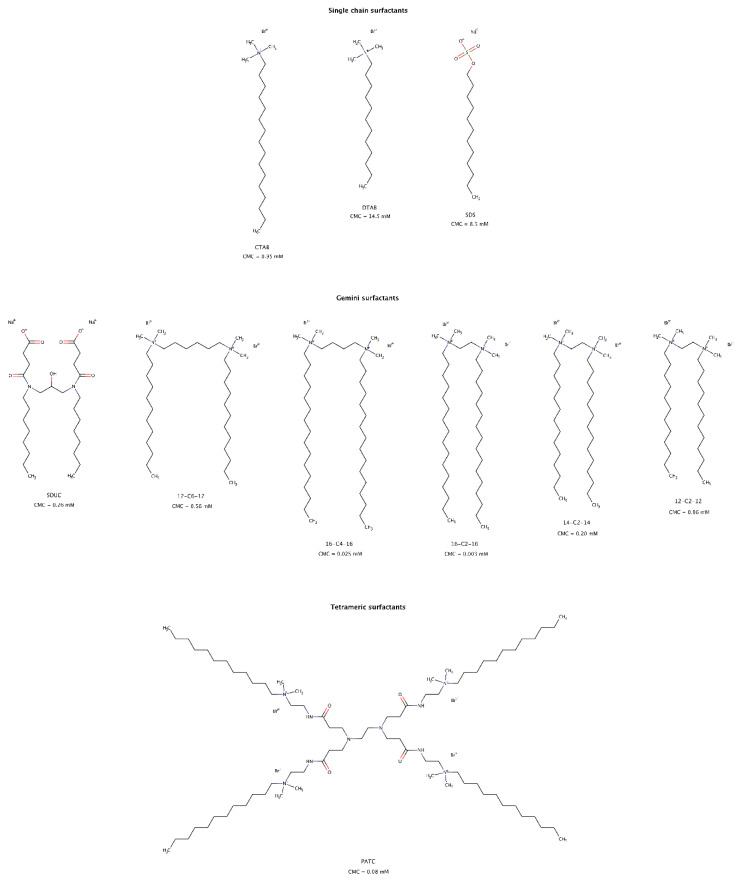
Chemical structures of surfactants capable of disrupting preformed amyloid fibrils. The CMC values shown were determined at 25 °C [200,204,205,206,207,208].

The use of surfactants to solubilize hydrophobic molecules in aqueous systems is well known and has multiple practical and industrial applications. More recently, the role of surfactants in modulating amyloid aggregation has also been studied. These amphiphilic molecules may favor β-rich structures and fibril formation, normally below the critical micelle concentration (CMC) [196,197], delay amyloid aggregation [197], inhibit amyloid fibril formation [198], and disrupt preformed fibrillar structures into soluble short fragments, usually above the CMC (Figure 13). These findings suggest that surfactants represent one more important category of molecules that can modulate both amyloid aggregation and disaggregation mechanisms. Several types of surfactants, namely cationic and anionic, single chain, gemini or dimeric, and tetrameric disassembled in vitro preformed fibrils of albumin [199,200], cystatin [201], and amyloid-β (Aβ) [202,203,204].

For fibrillar structures that were negatively charged at physiological conditions, the interaction with cationic surfactant micelles resulted in favorable electrostatic contacts facilitating its adsorption onto the fibrillar surface. Further rearrangement of detergent micelles allowed hydrophobic interactions between the “tails” of surfactant molecules and the hydrophobic regions of amyloid fibrils, as well as competition for the hydrogen bonds between β-strands, resulting in amyloid disassembly [199,201,202].

Gemini surfactants, consisting of two identical hydrophobic segments and two polar headgroups covalently linked by a spacer, have lower CMCs and a stronger self-aggregation ability than their single-chain counterparts (Figure 13). Micelles formed from gemini surfactants are powerful agents for disintegration of amyloid fibrils in consequence of their physico-chemical characteristics, i.e., positively bicharged hydrophilic heads, twin hydrophobic chains and strong self-aggregation abilities [200,201,202]. Longer hydrophobic tails have slightly larger disruptive effect than shorter tail surfactants [200].

Micelles and premicellar aggregates of the tetrameric surfactant PATC (N^1^,N^16^-didodecyl-7,10-bis(3-(2-(dodecyldimethylammonio)ethylamino)-3-oxoprpyl)N^1^,N^1^,N^16^,N^16^-tetramethyl-4,13-dioxo-3,7,10,14-tetraazahexadecane-1,16-diaminium tetrabromide) can effectively disassemble mature amyloid fibrils at a concentration as low as 0.02 mM, which is far below its CMC (Figure 13). However, gemini surfactants lose disassembly efficacy at this low concentration [203].

For anionic and single hydrophobic chain surfactants such as SDS (sodium dodecyl sulfate), micelles only had a small disaggregation effect on mature fibrils [199,201]. As observed for gemini detergents, single chain surfactants with longer alkyl chains had a greater effect on disaggregation of preformed fibrils [199].

Unlike PATC due to its strong self-association ability [207], gemini and single chain surfactants must be used at concentrations above their CMC to be effective in disrupting preformed amyloid fibrils. As a result, gemini surfactants are of greater interest than single chain surfactants due to their low CMCs and therefore potentially lower toxicity risks in in vivo experiments [209].

Surfactants are known to play a pivotal role in drug delivery systems. One of the main motivations in selecting cationic gemini surfactants to disassemble amyloid fibrils is because they can also serve as drug delivery carriers of other small molecule amyloid disaggregators [210]. On the basis of this dual action, low CMC surfactants may offer useful avenues in the formulation of pharmacological chaperones directed to amyloid clearance. 

## 3. Conclusions

The complexity of amyloid diseases, its progressive nature and staging, the involvement of multiple organs, and multiple clinical manifestations, requires several therapeutic strategies to be applied in single or combination therapies at different stages of the disease. A persistent central challenge regarding these pathologies is their early clinical diagnosis. Frequently, when a patient begins to exhibit clinical symptoms, extensive protein misfolding, aggregation, amyloid deposition and organ misfunction have already occurred. Thus, disaggregation-based approaches are particularly attractive because they can act directly on preformed aggregates and fibrils.

Since the beginning of the century, amyloid disassembly strategies have gained increasing attention. At first, several experimental in vitro approaches were explored to disfavor β-sheet formation and protein aggregation, and destabilize highly ordered and thermodynamically very stable fibrillar structures [1], namely ultrasonication, high and low temperatures, high pressure, and pH changes [211,212,213,214,215]. More recently, multiple studies using disaggregation agents have been performed in order to better understand disaggregation mechanisms not only using in vitro experiments, but also in vivo assays and even clinical trials.

Despite the differences in the primary structure of the misfolded polypeptide chains, chaperones of different types can disassemble fibrils of several proteins, suggesting a universal mechanism of action directed to the amyloid conformation. Amyloid fibrils share an extended β-sheet secondary structure, where individual β-strands are arranged perpendicularly to the main fiber axis, a structure known as cross-β motif. Additionally, amyloid aggregates and protofibrils tend to expose large hydrophobic surfaces.

Regarding the scaffolds of some chemical and pharmacological chaperones that act on amyloid, they also seem to share structural similarities. They usually contain planar hydrophobic moieties formed by aromatic rings or alkyl chains that interact directly with hydrophobic exposed regions in amyloid aggregates and fibrils. In addition, the hydrophobic aromatic cores may be coated with polar side chains, e.g., hydroxyl, carboxyl, and carbonyl groups, which interfere with the amyloid molecular organization by disrupting intra- and intermolecular hydrogen bonds of the β-sheet structure. In some cases, the number and the position of these hydrophilic groups seem to be critical for the disruptive activity of the chaperones.

The observed toxicity of oligomeric and prefibrillar molecular species may arise from the presence of hydrophobic groups on their surface. Due to the preferential hydrophobic interactions established between chaperones and amyloid structures, chaperones may suppress the toxicity of oligomeric intermediate amyloid species and promote the formation of amorphous aggregates or monomeric subunits, as reported in several instances. Nevertheless, the toxicity of oligomeric species makes amyloid disassembly a therapeutic strategy that deserves detailed study in order to control and prevent the formation of those toxic species. Furthermore, the release of monomeric species may initiate new aggregation events; thus, it is crucial to assure the native character of the newly formed monomers.

In summary, we reviewed a large set of currently known chemical and biochemical entities (chaperones) with the ability to disaggregate amyloids, their proposed mechanism of action, and their potential use as therapeutic solutions against amyloid diseases. There are still many unknowns concerning the clinical usefulness of these disaggregating agents, depending on the precursor protein, aggregation mechanism, affected organ(s) and disease. However, given the social and economic impact of these devastating diseases, it is of the utmost importance to have a detailed understanding of the nature of the disaggregating agents and their mechanism of action and continue to explore avenues in drug development that include the action of these agents.

## Data Availability

Data will be sent to interested researchers by request to the corresponding author.
